# Actors on the Scene: Immune Cells in the Myeloma Niche

**DOI:** 10.3389/fonc.2020.599098

**Published:** 2020-10-29

**Authors:** Patrizia Leone, Antonio Giovanni Solimando, Eleonora Malerba, Rossella Fasano, Alessio Buonavoglia, Fabrizio Pappagallo, Valli De Re, Antonella Argentiero, Nicola Silvestris, Angelo Vacca, Vito Racanelli

**Affiliations:** ^1^ Department of Biomedical Sciences and Human Oncology, University of Bari Medical School, Bari, Italy; ^2^ Department of Medical Oncology, IRCCS Istituto Tumori “Giovanni Paolo II” of Bari, Bari, Italy; ^3^ Bio-Proteomics Facility, Department of Translational Research, Centro di Riferimento Oncologico di Aviano (CRO) IRCCS, Aviano, Italy

**Keywords:** multiple myeloma, microenvironment, immune cells, immune checkpoints, immunotherapy

## Abstract

Two mechanisms are involved in the immune escape of cancer cells: the immunoediting of tumor cells and the suppression of the immune system. Both processes have been revealed in multiple myeloma (MM). Complex interactions between tumor plasma cells and the bone marrow (BM) microenvironment contribute to generate an immunosuppressive milieu characterized by high concentration of immunosuppressive factors, loss of effective antigen presentation, effector cell dysfunction, and expansion of immunosuppressive cell populations, such as myeloid-derived suppressor cells, regulatory T cells and T cells expressing checkpoint molecules such as programmed cell death 1. Considering the great immunosuppressive impact of BM myeloma microenvironment, many strategies to overcome it and restore myeloma immunosurveillance have been elaborated. The most successful ones are combined approaches such as checkpoint inhibitors in combination with immunomodulatory drugs, anti-monoclonal antibodies, and proteasome inhibitors as well as chimeric antigen receptor (CAR) T cell therapy. How best to combine anti-MM therapies and what is the optimal timing to treat the patient are important questions to be addressed in future trials. Moreover, intratumor MM heterogeneity suggests the crucial importance of tailored therapies to identify patients who might benefit the most from immunotherapy, reaching deeper and more durable responses.

## Introduction

Multiple myeloma (MM) is a malignant plasma cell disease mainly located in the bone marrow (BM) in multiple ‘niches’. These provide a microenvironment that promotes tumor survival and progression. Within BM niches, normal and tumor plasma cells can survive for years, even for decades. Moreover, the observation that tumor plasma cells do not grow and expand when cultured alone suggests the huge resilience of these cells within the BM microenvironment (1). The BM milieu consists of a cluster of cells such as immune cells, stromal cells, endothelial cells (ECs), and bone cells, soluble factors (cytokines, chemokines, and growth factors), and non-cellular matrix (2). It is highly vascularized by blood vessels and is a part of the lymphocyte re-circulation network. Cells re-circulating into and out of the BM have the potential to regulate tumor plasma cell growth and progression through a composite array of indirect and direct interactions involving cytokines as well as surface (3) and soluble molecules (4). In this context, the immune system plays a central and multifaceted role.

**Figure 1:**
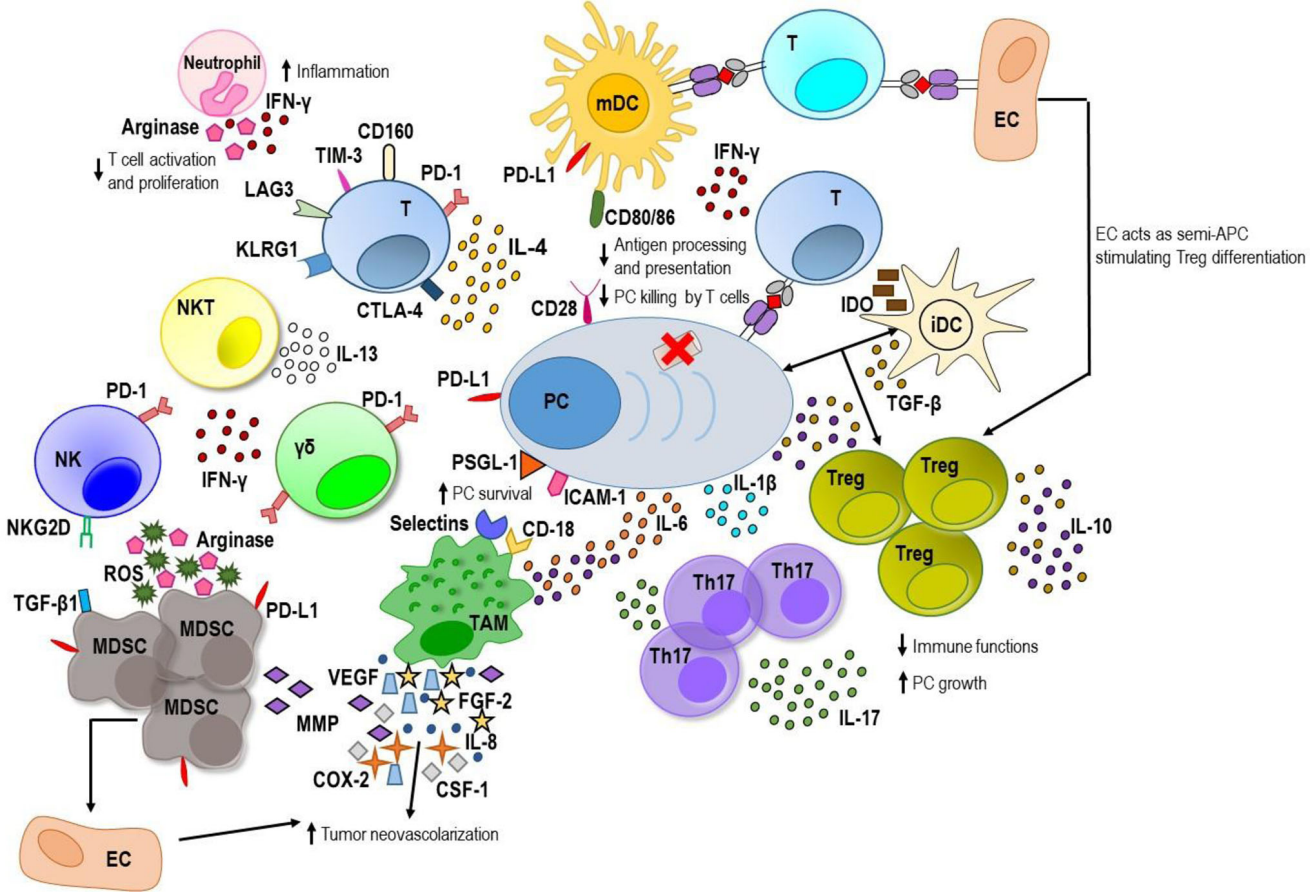
The MM BM microenvironment. On one hand, innate and adaptive immune cells are able to recognize myeloma plasma cells (PC) and generate a weak immune response against tumor. Mature dendritic cells (mDCs) activate tumor-specific T cells that along with natural killer (NK), NKT and gamma delta (*γδ*) T cells produce low amount of interferon (IFN)-*γ*. On the other hand, myeloma PCs are able to promote an immunosuppressive microenvironment. They produce immunosuppressive factors including transforming growth factor (TGF)-*β*, interleukin (IL)-10 and IL-6. PC–immature DC (iDC) interaction stimulates TGF-*β* production by DC inducing T regulatory (Treg) proliferation with enhancement of levels of TGF-*β* and IL-10. Immature DCs produce also indoleamine 2,3-dioxygenase (IDO) that causes anergy in activated T cells. The latter exhibits exhaustion markers such as programmed cell death-1 (PD-1), cytotoxic T lymphocyte antigen-4 (CTLA-4), T cell immunoglobulin-3 (TIM-3), and lymphocyte-activation gene 3 (LAG3), and high levels of the senescence markers killer-cell lectin like receptor G1 (KLRG1) and CD160. PD-1 is greatly expressed also by *γδ* T cells and NK cells and interacts with its ligand, programmed death ligand 1 (PD-L1), expressed by myeloma PC, DC, and myeloid derived suppressor cells (MDSCs) downregulating immune response. Myeloma PC–mature DC interaction, involving the CD28 receptor and the CD80/CD86 ligands respectively, downregulates proteasome subunit expression in tumor PC and decreases the processing and presentation of tumor antigens thus reducing myeloma PC recognition by cytotoxic CD8^+^ T cells. Myeloma PC-tumor-associated macrophage (TAM) interaction involving P-selectin glycoprotein ligand 1 (PSGL-1) and intercellular adhesion molecule-1 (ICAM-1) on myeloma PC and E/P selectins and CD18 on TAM confers multidrug resistance to MM PC. Within myeloma niche, TAMs release great amount of IL-6 and IL-10 and contribute to MM-associated neovascularization by vasculogenic mimicry and indirectly by secreting vascular endothelial growth factor (VEGF), IL-8, fibroblast growth factor-2 (FGF-2), metalloproteinases (MMPs), cycloxygenase-2 (COX-2), and colony-stimulating factor-1 (CSF-1). Neutrophils release high amount of IFN-*γ* that supports their promotion of pro-inflammatory and survival signals within the plasma cell niche and produces arginase that inhibits T cell activation and proliferation. MDSCs also produce high amounts of arginase and reactive oxygen species (ROS) that contribute to T cell suppression, induce anergy of NK cell through membrane-bound TGF-*β*1 and promote tumor angiogenesis by MMP secretion or direct differentiation into endothelial cells (ECs). Furthermore, ECs can act as semi-professional antigen presenting cells (APCs) stimulating a regulatory tumor-specific T cell population. Within BM, the elevated levels of IL-6, TGF-*β*, and IL-1*β* promote T helper IL-17-producing (Th17) cell polarization which release high levels of IL-17 favoring MM plasma cell growth and inhibiting immune system.

A multistep development model indicates that MM progresses from a stable premalignant plasma cell clonal expansion termed monoclonal gammopathy of undetermined significance (MGUS). This asymptomatic preneoplastic condition is characterized by a perfect equilibrium between tumor and immune system which allows disease to remain stable and does not develop to MM. Immune cells control, but not eliminate MGUS plasma cells. These findings suggest that malignant transformation depends not only on the features of the tumor cells themselves but also on the surrounding microenvironment and its effects on tumor cells. Complex cancer–immune system interactions generate both pro- and anti-tumor effects whose balance can be altered in favor of an immunosuppressive environment which promote tumor progression (5, 6). On one hand, innate and adaptive immune cells are able to detect tumor plasma cells; tumor-specific cytotoxic T cells can be found in the BM of MGUS and MM patients (7, 8). On the other hand, tumor plasma cells have the ability to promote a tolerant microenvironment and the activation of immunosuppressive mechanisms to counteract effective immune responses. These include impairment of antigen processing and presentation, and T cell response, NK and NKT cell dysfunctions, local recruitment, expansion and activation of immune suppressor cells like T regulatory cells (Tregs) and myeloid derived suppressor cells (MDSCs), and differentiation of the protumoral tumor-associated macrophages and Th17 cells (9–11) (**Figure 1**).

Here we describe interactions between BM tumor plasma cells and different immune cells and provide an overview of the current knowledge on immunotherapeutic strategies.

## Myeloma Plasma Cell Immunogenicity

The hallmark of MGUS and MM plasma cells is the production and the surface expression of a monoclonal immunoglobulin (Ig) carrying unique antigenic (idiotypic or Id) determinants in the variable heavy (VH) (12). Thus, the Ig idiotypic structure is a tumor-specific antigen of the myeloma cell clone, distinct from normal cells or normal plasma cells that can be presented as whole molecule on the cell surface or as peptides in the groove of the of major histocompatibility complex (MHC) molecules (13, 14).

Several studies have described idiotype-specific cytotoxic T lymphocytes in MM patients with the capacity to lyse autologous primary tumor plasma cells. Many potential T cell epitopes have been identified within the tumor-derived Ig-VH region, nonetheless, the majority of them didn’t trigger high affinity T cell responses (15). Two peptide prediction algorithms, BIMAS and SYFPEITHI, have also confirmed the poor immunogenicity of human idiotypes with a low binding half-life (BIMAS) and a low/intermediate score (SYFPEITHI) on most T cell interaction human leukocyte antigen (HLA) modules (16, 17). Additionally, idiotypic vaccination in MM has been examined in clinical trials where immunologic responses occurred in <50% of patients, and clinical responses have been infrequent (18).

Within the universal tumor antigens, many myeloma-associated antigens (*e.g.* human telomerase reverse transcriptase (hTERT) (19), surviving (20), new york esophageal squamous cell carcinoma 1 (NY-ESO1) (21) mucin-1 (MUC-1) (22), junctional adhesion molecule-A (JAM-A) (23, 24) and the receptor for hyaluronic acid-mediated motility (RHAMM) (25) have been identified as targets recognized by T lymphocytes and used in many vaccination strategies, but in most cases failed to produce clinically meaningful responses. However, many obstacles need to be overcome. The most important one is the myeloma plasma cell escape of tumor-specific immune response. Our group demonstrated that the binding of CD28 expressed on myeloma plasma cells with its ligands CD80/CD86 expressed on BM dendritic cells (DCs) results in downregulation of the expression of proteasome subunits, alteration of the antigen repertoire displayed on myeloma plasma cell surface, and reduced recognition of tumor plasma cells by cytotoxic CD8^+^ T cells (10).

## Tumor-Specific Cytotoxic CD8^+^ T Cells

The mechanisms underlying MGUS to MM progression are incompletely understood. Tumor plasma cell specific CD8^+^ T cells have been detected in both MGUS and MM patients (8, 26, 27), thereby establishing that there is no tolerance to plasma cell tumors. Nonetheless, in MM, myeloma plasma cell proliferation is not counteracted by CD8^+^ T cells. These observations have rekindled interest in the immunosurveillance mechanisms of tumor growth (28). Although MM plasma cells do not significantly differ from their premalignant MGUS precursors with respect to cytogenetic abnormalities (29, 30) and gene expression profiles (31), CD8^+^ T cells have been uncovered to fail to limit the clonal expansion of tumor plasma cells in MM. Our studies have shown that malignant transformation of plasma cells is associated with altered expression of HLA class I antigen processing presenting machinery (APM) components. These alterations are detectable *ex vivo*, occur at the transcriptional level, and, in some cases, are enhanced by IL-6, an essential MM cytokine. For some APM components, changes correlate with the extent of the plasma cells’ lysis by CD8^+^ T cells and with variations in the serum level of the M component in MGUS patients (8). Downregulation of proteasome subunits, in particular, is higher in plasma cells from MM patients than in those from MGUS patients and MM plasma cells are less readily lysed by autologous, *in vitro*-expanded cytotoxic CD8^+^ T cells than are MGUS plasma cells. This difference in cytotoxicity is evident at the epitope level and is not due to the intrinsic features of CD8^+^ T cells, given that no difference is observed when CD8^+^ T cells are tested against HLA-matched target cells that are not plasma cells (8). These findings support the hypothesis that proteasome subunit downregulation decreases expression of tumor antigen peptides on tumor plasma cells, enabling them to evade CD8^+^ T cell recognition and killing (10). Moreover, these alterations in the expression of APM components are specific of each premalignant and malignant plasma cell clone, suggesting that the myeloma-specific T cell response can differ from one patient to another. Indeed, CD8^+^ T cells isolated from MGUS and MM patients can be activated *ex vivo* by DC loaded with autologous but not allogeneic tumor lysates (7, 27, 32). The finding that the impairment of T cell response is restricted to myeloma antigens is supported also by the absence of a clinical T cell immunodeficiency. Myeloma patients show an appropriate T cell immunity against external antigens and do not show an increased incidence of mycobacterial infections or virus associated second malignancies (33).

Some shared antigens have been identified as targets of a spontaneous immune response in MGUS but not MM patients suggesting the capacity of the immune system to recognize premalignant lesions. For instance, the clonogenic CD138^−^ compartment in MGUS patients expresses SOX2, an embryonal stem cell protein involved in the tumor-initiating potential and self-renewal of tumor cells. The expression of this antigen identifies potential MM progenitors and detection of anti-SOX2 T cells is associated with an improved clinical outcome in patients with asymptomatic plasma cell disorders. SOX2 is also expressed by CD138^+^ cells in patients with active MM, who do not develop anti-SOX2 immunity (34).

Recent evidence also indicates that MM-specific cytotoxic CD8^+^ T cells do not express CD28 and express low levels of programmed cell death-1 (PD-1), cytotoxic T lymphocyte antigen-4 (CTLA-4), lymphocyte-activation gene 3 (LAG3), and T cell immunoglobulin-3 (TIM-3) (35). These characteristics further strengthen the idea that MM-specific CD8^+^ T cells are not anergic or exhausted. Instead, they seem to be “senescent” given that they express high levels of CD57, CD160, and killer-cell lectin like receptor G1 (KLRG1), do not express CD27 and CD28, and display weak proliferation after antigen stimulation (35). So basically, MM-specific CD8^+^ T cells appear late-differentiated and suspended in a hypo-responsive, non-proliferative state. Interestingly, this state would be telomere-independent and potentially reversible (35) since MM-specific CD8^+^ T cells have normal-for-age telomere lengths and long-surviving MM patients retain cytotoxic T cell clones with conserved proliferative capacity (36).

## CD4^+^ T Helper Cells

The role of CD4^+^ T helper cells in MM is still unclear; controversial data may be justified by differences between BM and peripheral blood, or by different quantification method (absolute count *versus* percentage), or by changes in Th1/Th2 polarization during the course of the disease. While some authors reported an altered Th1/Th2 balance strongly supported by IL-6, with increased production of Th2 cytokines, such as IL-10 and IL-4 and decreased production of Th1 cytokines, such as IL-2 and IFN-γ (37, 38), others described an elevated Th1/Th2 ratio in MM patients at diagnosis and in refractory phase, pointing towards a close relation to the clinical features (39–41).

Furthermore, increased levels of PD-1 on CD4^+^ cells have been observed in MM patients with persistent minimal residual disease (MRD) and at relapse compared with T cells of first diagnosed MGUS and MM patient (42). BM myeloma PD-1 expressing CD4^+^ T cells interact with plasma cells and DCs that display on their surface programmed death ligand 1 (PD-L1) promoting T cell suppression and MM progression (43).

## T Helper 17 Cells

Elevated levels of interleukin (IL)-6, transforming growth factor (TGF)-*β*, and IL-1*β* in myeloma BM microenvironment promote T helper IL-17-producing (Th17) cell polarization with consequent increased of IL-17 levels in BM and peripheral blood of MM patients (36, 44–46). IL-17 induces myeloma cell growth and colony formation *via* IL-17 receptor and inhibits Th1 immune response (45). The amount of Th17 cells in the BM positively correlates with clinicopathological characteristics in MM, like clinical tumor stage, serum lactate dehydrogenase concentration, and serum creatinine concentration (46). In addition, IL-17 plays a role in osteoclast-mediated lytic bone disease (44). Recently, the existence of a direct immunological link between the gut and the BM in MM involving Th17 cells has been proposed. Using a Vk*MYC mouse model, it has been provided that the gut microbiota induces the differentiation of Th17 cells in the gut that are able to migrate to the BM, where they promote MM progression (47). In the BM, IL-17 activates also eosinophils involved in plasma cell homing to the BM and in their accumulation in the BM niche (47, 48).

## Regulatory T Cells

There is considerable controversy regarding regulatory T (Treg) cell frequency and function in MM due to their source (peripheral blood *versus* BM), differences in assays, purification techniques, and markers used to identify these cells. Treg population is described as reduced and/or dysfunctional (39, 49, 50) or increased and/or functional (36, 51–57) in MM patients compared to MGUS patients or normal controls. Moreover, the increased frequency of CD4^+^ and CD8^+^ Treg cells in MM patients correlated with the active phase (54) and a reduced survival (55, 58). MM plasma cells can directly induce functional Treg in a contact dependent manner acting as immature and tolerogenic antigen presenting cells (APCs) (54) as well as in an APC independent manner by the expression of the inducible T cell co-stimulator ligand (ICOSL) (59). It is possible that the mutual and dynamic interactions among cells of the BM microenvironment along with cytokine release modulate the frequency and the suppressive activity of Treg cells. In coculture experiments, cytokines such as IL-10 and TGF-*β* and human myeloid immature DCs are the most efficacious for induction and expansion of Treg population (60, 61). Tumor cell–immature DC interaction stimulates TGF-*β* production by DC inducing Treg proliferation (62). Our study demonstrated that EC can act as semi-professional APC stimulating a regulatory tumor-specific CD8^+^ T cell population with suppressive function within BM of MM patients (11). Moreover, human leukocyte antigen G (HLA-G)^+^ T cells with an inhibitory activity comparable to natural Treg can be generated in BM of MM patients after tumor plasma cell–T cell interaction by trogocytosis of immunosuppressive molecules such as HLA-G (63).

## *γδ* T Cells

BM MM microenvironment is extremely immunosuppressive and greatly influences gamma delta (*γδ*) T cells. Indeed, BM derived V*γ*9V*δ*2 T cells, the main subset of *γδ* T cells, become more dysfunctional than those isolated from the peripheral blood of MM patients. The functional exhaustion of BM V*γ*9V*δ*2 T cells occurs early during disease progression and does not disappear in clinical remission. Upregulation of PD-1 expression on *γδ* T cells is already found in MGUS patients and persists in the remission phase and further increases in the relapse (64). One possible explanation is the great concentration of phosphoantigens in the tumor microenvironment and the consequent prolonged TCR engagement. High amounts of isopentenyl pyrophosphate, the prototypic phosphoantigen recognized by V*γ*9V*δ*2 T cells *via* TCR, are produced by both BM myeloma plasma cells and stromal cells leading to chronic TCR engagement, upregulation of PD-1 expression, and functional exhaustion of *γδ* T cells (65). Moreover, many cell subsets including MM plasma cells, myeloid-derived suppressor cells (MDSCs), regulatory T cells (Tregs), and BM-derived stromal cells are implicated in V*γ*9V*δ*2 T cell hampering through the excessive expression of immune checkpoints (ICP)/ICP-ligands (65).

## Dendritic Cells

The role of DC in MM progression is controversial. Some investigators reported impaired functionality and phenotypic profile, while others found that these cells are normal (9). We have demonstrated that BM DCs are functional and play a dual, but opposing role in MM. DCs are concentrated in the BM during the MGUS-to-MM progression and interact with both T cells and myeloma plasma cells. On one hand, DCs are able to uptake apoptotic myeloma plasma cells, mature and process myeloma antigens, cross-present them and successfully activate myeloma-specific BM-infiltrating CD8^+^ T cells. On the other hand, by using their surface CD80/86 molecules, DCs interact with non-apoptotic tumor plasma cells by the CD28 receptor that is upregulated on their surface, promoting a downregulation of proteasome subunit expression and a consequent escape of myeloma plasma cells from CD8^+^ T cell recognition and killing (10). Moreover, plasmacytoid DCs promote tumor plasma cell growth, survival, and drug resistance (66) and express high surface levels of programmed death-ligand 1 (PD-L1) conferring T cell and NK cell immune suppression by engaging ICP *via* PD1-PD-L1 signaling axis (67, 68). Myeloid CD141^+^ DCs also express PD-L1, and the proportion of these cells correlate with the percentage of PD-L1^+^ plasma cells, suggesting that both cell subsets support anti-tumor T cell response inhibition in MM (68). DCs can also indirectly favor the osteoclastogenesis process by inducing Th17 cell expansion in BM myeloma microenvironment (69) followed by IL-17 accumulation, a potent pro-osteoclastogenic factor (70).

## Macrophages

Tumor-associated macrophages (TAMs) constitute an abundant component of myeloma microenvironment that induce myeloma plasma cell survival through both contact-dependent and -independent mechanisms. For instance, a direct physical interaction involving E/P selectins and CD18 on macrophages and P-selectin glycoprotein ligand 1 (PSGL-1) and intercellular adhesion molecule-1 (ICAM-1) on myeloma cells protects plasma cells from drug-induced apoptosis (71–73). Within myeloma niche, after interaction with BM-derived mesenchymal stromal cells, TAMs acquire a secretory profile characterized by a great production of IL-6 and IL-10 and poor production of IL-12 and TNF-α, providing a suitable milieu for myeloma plasma cell growth (74). TAMs also contribute to MM-associated neovascularization by vasculogenic mimicry and indirectly by secreting a wide range of proangiogenic factors, such as vascular endothelial growth factor (VEGF), IL-8, and fibroblast growth factor-2 (FGF-2) as well as metalloproteinases (MMPs), cycloxygenase-2 (COX-2), and colony-stimulating factor-1 (CSF-1). Moreover, they resemble M2-like macrophage population with a reduced cytotoxic capacity for tumor cells and a decreased antigen-presenting capability (75).

A very recent single-cell RNA sequencing study revealed that mature CD14^+^ monocytes/macrophages lose HLA class II surface expression as early as in the MGUS phase resulting in T cell suppression (76).

## NK Cells

Natural killer (NK) cell differentiation, activation, and cytotoxic ability are strongly compromised during MM progression (77). BM myeloma plasma cells from early-stage patients display low levels of MHC class I molecules and high levels of MHC class I related chain A (MICA) and are readily recognized by NK cells (78). Nevertheless, elevated numbers of NK cells in the BM and blood of MM patients were associated with worse prognoses (79). Myeloma cell recognition and killing by NK cells involve a broad array of activating receptors including the natural killer group 2D (NKG2D), DNAX accessory molecule-1 (DNAM-1), and the natural cytotoxicity receptors (NCR) NKp46, NKp30, and NKp44 (78, 80). Changes in the expression of these NK receptors and NK cell receptor ligands have been observed in BM samples of MGUS and MM patients, suggesting a role of NK cell dysfunction during MGUS-to-MM progression (81). In addition, following an extensive interaction with cytotoxic T and NK cells, myeloma plasma cells obtained from patients with active disease exhibit the MHC class I^bright^/MICA^dim/−/^CD95^dim/−^ immunophenotype that compromises NK cell function (78, 82, 83). Likewise, the number of effector NK cells localized within the BM progressively decreases during MM growth and correlates with reduced BM NK cell degranulation in MM-bearing mice (84). Moreover, MICA shedding from the surface of myeloma plasma cells may promote downregulation of NKG2D expression on the surface of NK cells weakening the NK-mediated anti-tumor response (83, 85). Defective NK cell functions can be also explained by PD-1 expression on NK cells of MM patients that interact with its ligands PD-L1 on tumor plasma cells downregulating NK cell function (86). Also, the release of soluble factors in the BM microenvironment can influence NK cell activity. For instance, an inflammatory milieu rich in IFN-*γ* secreted by immune cells strongly increases PD-L1 expression (87). In addition, primary myeloma plasma cells express high levels of HLA-E molecules which bind to the inhibitory NK cell receptor NKG2A hampering NK cell effector functions (80, 88–90).

## NKT Cells

NKT dysfunction has been recognized as potentially important in disease predisposition and progression (91). A progressive decrease of NKT cells and a loss of both peripheral blood and BM NKT cell activity in MM patients have been described by many groups, with disease progression correlating with a reduction of IFN-*γ* production by NKT cells (26, 92, 93). Likewise, a loss of CD1d expression by myeloma plasma cells has been demonstrated during disease progression with consequent dysfunction of NKT cells (26, 92, 94, 95).

A recent study has demonstrated an enrichment of inflammation-associated lysophosphatidylcholine molecules in MM patient serum compared with healthy donors alongside with an increase of frequency of lisophosphatidylcholine-recognizing CD1d-restricted type II NKT cells. These cells release high amounts of the IL-13, an immunosuppressive cytokine involved in tumor-promoting inflammation and angiogenesis, thus supporting their role in disease progression (96). Furthermore, type II NKT cells may also promote plasma cell differentiation and play a role in the initiation of MM (97, 98).

## Myeloid-Derived Suppressor Cells

In humans, two main subsets of myeloid derived suppressor cells (MDSCs) with the same level of suppressive activity can be identified based on CD14 positivity, granulocyte-MDSCs (G-MDSCs) that are CD11b^+^CD14^−^CD33^+^CD15^+^HLA-DR^−/low^ and monocytic-MDSCs (M-MDSCs) that are CD11b^+^CD14^+^CD33^+^HLA-DR^−/low^ (99). The involvement of these subsets in the pathogenesis of MM is still not clear. Several studies found a significant increase in G-MDSCs in the peripheral blood and BM of newly diagnosed, relapsed, and relapsed/refractory MM patients compared with healthy donors (100–102), while others described an increase of M-MDSCs in first diagnosed and relapsed MM patients compared with those in remission and healthy donors (103, 104). Moreover, the level of M-MDSC correlates with disease progression (104).

Because of their capacity to suppress T cell-mediated immunity, MDSCs play an important role in favoring tumor escape from immunosurveillance (101, 102). MDSCs secrete high amount of arginase which sequestrates L-arginine, an essential amino acid for T cell activity (105). Moreover, MDSCs can inhibit T cell receptor by nitrosylation and reactive oxygen species release (106) and express on their surface high levels of PD-L1 which can interact with PD-1 express on T cells (64).

In addition, MDSCs induce Treg differentiation through TGF-*β*-dependent and -independent mechanisms involving CD40 or IL-10 and IFN-*γ*, respectively (100, 106), induce NK cell anergy through membrane-bound TGF-*β*1 (107, 108), promote tumor angiogenesis by MMP-9 secretion or direct differentiation into EC (109), and stimulate tumor growth through the release of cytokines and growth factors (101).

Using immunocompetent mouse models, it has been demonstrated that MDSC immunosuppression occurs early in MM disease; MDSCs accumulated in the BM of mice as early as one week after tumor inoculation and when these mice were engineered to lose their ability to accumulate MDSC, growth of MM plasma cells was significantly reduced confirming the critical role of MDSC accumulation at early stages of MM progression (102).

## Neutrophils

Neutrophils are essential for clearance of extracellular pathogens, as they effectively fight them by releasing cytotoxic granules, toxic enzymes, inflammatory mediators, and reactive oxygen species (110, 111). Thanks to a large number of integrins and molecules expressed on their surface, neutrophils can establish interactions with other immune cells (*e.g.*, T, B, and NK cells, monocytes, macrophages, DCs), can act as weak antigen presenting cells, promote angiogenesis and inflammation, and regulate hematopoiesis (112). In MM, as a consequence of BM infiltration by tumor plasma cells, functional defects of neutrophils including reduced lysozyme activity and increased secretion of the amino acid degrading enzyme, arginase, have been described (113, 114). An involvement of neutrophils in immune suppression *via* IFN-*γ* signaling has been also revealed since the early asymptomatic phase of MGUS (115). Specifically, Romano et al. (115) have demonstrated that neutrophils from MGUS and MM are chronically activated because of increased signaling through IFN-*γ* and Toll-like receptors that trigger a chronic inflammatory response *via* STAT protein activation. Compared with neutrophils from healthy patients, neutrophils from MGUS and MM patients show immunosuppressive features. They display an impairment in the FC-*γ*-receptor I (CD64) mediated phagocytosis under control of IFN-*γ* and increased secretion of arginase-1, target of STAT proteins (116–118), resulting in inhibition of T cell activation and proliferation (115). Furthermore, during MGUS-to-MM progression, neutrophils progressively enhance the production of IFN-*γ* in response to MM soluble factors resulting in increased autophagy flux and JAK-2/STAT3 pathway activation, which support their promotion of pro-inflammatory and survival signals within MM niches (119).

In addition, the neutrophil to lymphocyte ratio (NLR) at diagnosis or after 100 days from autologous stem cell transplantation (SCT) can predict outcome in newly diagnosed MM patients treated upfront with novel agents (120–122). Interestingly, NLR could be combined with international staging system (ISS) to better evaluate the risk profile of non-elderly (<65 years) MM patients, to identify patients with poor outcome, and to personalize MM therapy (121).

## Immune Checkpoints in Multiple Myeloma

The main ICP pathways CTLA-4 and PD-1/PD-L1 have emerged as major immune escape mechanisms in MM. These pathways are crucial in the physiological setting for maintaining the immune equilibrium after the initial T cell response and preventing over-activation of the immune system and tissue damage. Tumor cells upregulate these biologic mechanisms of tolerance and exploit them to elude host immunity (123). Regarding MM, contradictory results exist in this field, due mainly to the different analyzed sources (peripheral blood *versus* BM) suggesting a fundamental role of the local milieu in the regulation of immune ICP cell expression.

Several studies have found an increased number of CTLA4^+^ Treg cells in the BM of MM patients compared with MGUS patients and healthy donors (52, 56, 124), with a correlation between the proportion of cells simultaneously positive for CTLA4 and Foxp3 and the disease stage (54).

PD-1 expression is increased on NK and *γδ* T cells isolated from MM patients and correlates with loss of effector cell function (64, 86). CD4^+^ and CD8^+^ T cells express low level of PD-1 in MGUS and newly diagnosed MM patients, suggesting that downregulation of their effector function is partly due to senescence rather than PD-1 mediated exhaustion (35, 124, 125). Paiva et al. have reported increased PD-1 expression levels on CD4^+^ and CD8^+^ T cells only in relapsed or relapsed/refractory MM and in patients with a minimal residual disease (42).

PD-L1 is greatly expressed on plasma cells obtained from MM patients with active, relapsed, and refractory disease, whereas low expression has been found on plasma cells from MGUS patients or healthy donors, suggesting that PD-L1 expression is associated with MM progression and drug resistance (42, 68, 126–128). Moreover, soluble factors such as IFN-*γ*, IL-6, and indoleamine 2,3-dioxygenase (IDO), detected at high level in myeloma BM microenvironment, upregulate the expression of PD-L1 on myeloma plasma cells (126, 127, 129). PD-L1 is also expressed by other cells of myeloma BM microenvironment, including plasmacytoid DC, NK cells, and MDSCs, according to their immunoregulatory functions (67, 68, 86, 100, 127).

## Immunosuppressive Factors

Along with the crosstalk between tumor plasma cells and BM niche cells, a high concentration of immunosuppressive factors including TGF-*β*, IL-10, IL-6, and prostaglandin E2 in the MM BM microenvironment promotes tumor propagation and survival and at the same time generates great immune dysfunction (130). In addition, the cellular contact of myeloma plasma cells with BM immature DCs, through CD47–thrombospondin-1 interaction, leads to spontaneous DC fusion and trans-differentiation into osteoclasts (131, 132), which, beside their role in bone lesions, promote suppressive immune BM microenvironment inducing T cell apoptosis by the overexpression of ICP molecules and the release of IDO and APRIL (129). Moreover, IDO causes anergy in activated T cells, induces them to become Treg, and generates a nutritionally depleted niche favoring survival of myeloma cells which have a low proliferative index and are less sensitive to tryptophan depletion (133); APRIL enhances PD-L1 expression on MM cells supplying immune suppression (129). Simultaneously, the establishment of a chronic inflammatory status contributes also to disease progression (134). Increased levels of inflammatory cytokines, such as IL-1, IL-6, IL-12, IL-15, IL-17, IL-18, IL-22, IL-23, TNF-*α*, and IFN-*γ* have been revealed in BM serum of MM patients (135), and an eight-gene signature (IL-8, IL-10, IL-17, CCL3, CCL5, VEGFA, EBI3, and NOS2) involved in B-cell inflammation has been described able to distinguish the different phases of disease progression (MGUS/smoldering/symptomatic-MM) with 84% accuracy (134). Moreover, inflammation can lead to high levels of bioactive lipids, such as several species of lysophosphatidylcholine, which can bind to CD1d molecules resulting in dysregulation of lipid-reactive immune cells, activation of CD1d-restricted type II NKT cells, and production of high amount of the immunosuppressive cytokine IL-13 (96).

## Targeting Immune System as an Effective Approach to Treat Multiple Myeloma

**Figure 2:**
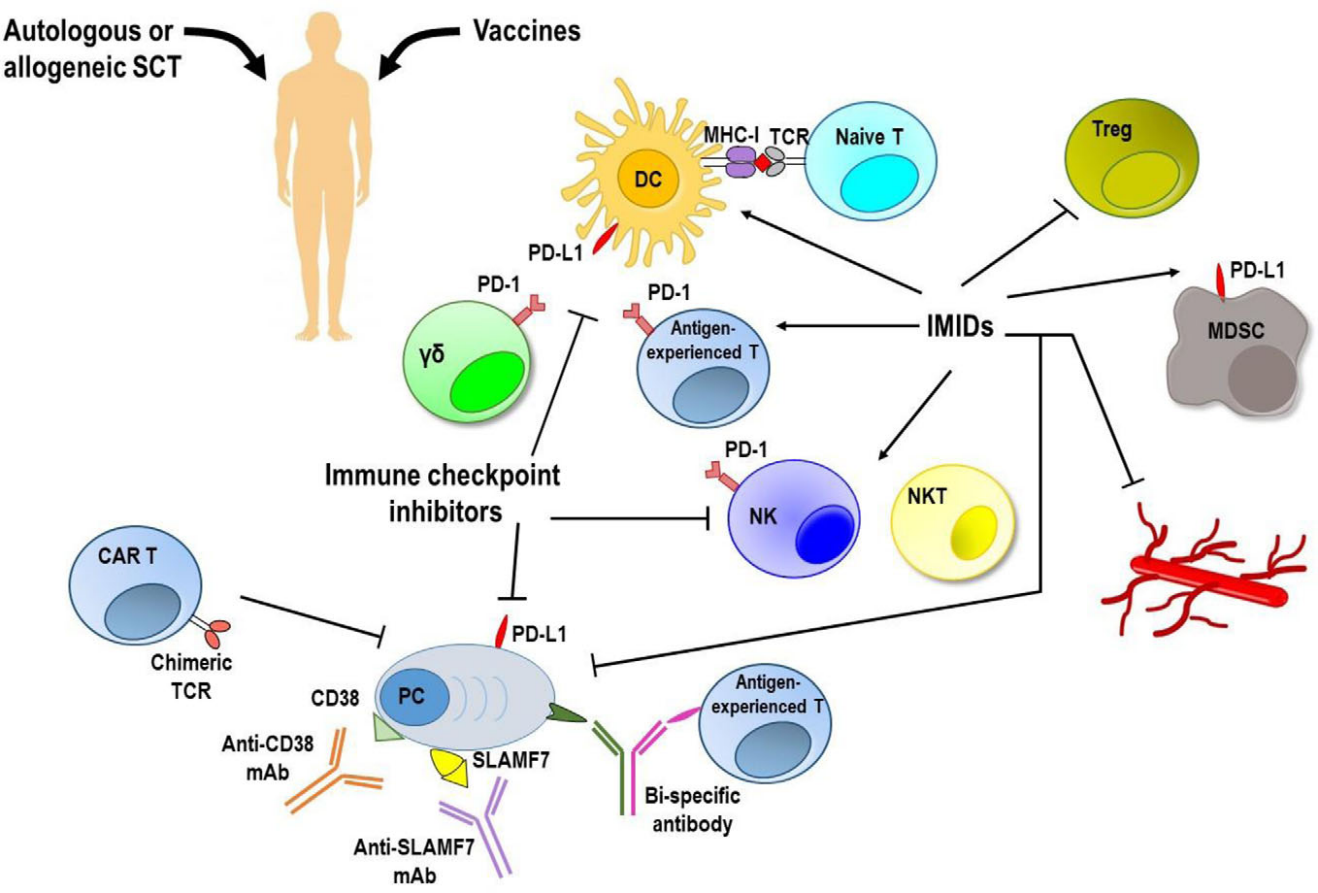
Targeting immune system to induce anti-MM responses. MM immunosuppressive microenvironment remains the major hurdle to achieve a long lasting response along with low toxicity. Vaccination strategies have shown no clear clinical efficacy. Autologous and allogeneic stem cell transplantation (SCT) following myeloablative treatment allows introduction of a new immune system, but generates a very weak anti-tumor immune response. Immune checkpoint inhibitors, immunomodulatory drugs (IMiDs), and monoclonal antibodies (mAbs) used as single agents provided unsatisfactory results. Immunotherapy with adoptively transferred chimeric antigen receptor (CAR) T cells and new bi-specific antibodies are currently being tested in clinical trials, and initial results have been encouraging. Moreover, newer approaches based on the combination of immunotherapeutic strategies are achieving promising results with acceptable safety and durable responses. DC, dendritic cells; *γδ*, gamma delta T cells; MDSC, myeloid derived suppressor cells; MHC-I, major histocompatibility complex-class I; NK, natural killer cells; NKT, natural killer T cells; PC, plasma cells; PD-1, programmed cell death-1; PD-L1, programmed death ligand 1; SLAMF7, family member 7 of the signaling lymphocytic activation molecule; TCR, T cell receptor; Treg, regulatory T cells.

Considering the great immunosuppressive impact of BM myeloma microenvironment, many strategies to overcome it and restore myeloma immunosurveillance have been elaborated (**Figure 2**). Autologous SCT following myeloablative treatment allows the introduction of a new immune system and has significantly contributed to improve survival of MM patients in the last 15 years (136). Unfortunately, the graft *versus*-myeloma (GvM) response is usually weak and most patients relapse. An alternative is the adoptive therapy with BM infiltrating lymphocytes enriched in myeloma-specific T cells that enhances the anti-tumor immunity, but has a poor durability of the clinical response (137), or the allogeneic SCT which provides a new T cell repertoire, triggers a potent GvM response, but it is limited by the high transplant-related mortality (138).

The emergence of ICP blockade therapies over the last decade raised great interest also in MM. Despite at first, *in vitro* and *in vivo* studies showed that PD-1/PD-L1 blockade enhanced T and NK cell mediated anti-myeloma immune responses (42, 64, 86, 139–141) suggesting that ICP inhibition may be a promising therapeutic strategy against MM, clinical trials have provided unsatisfactory results (125). A possible explanation is that myeloma-specific T cells have an anergic or senescent phenotype rather than an exhausted phenotype, a prerequisite for the success of ICP blockade therapies.

Current lines of evidence indicate that the senescent phenotype could be reversed by immunomodulatory drugs (IMiDs), thalidomide, and its analogs lenalidomide and pomalidomide, or histone deacetylase inhibitors (35). Besides their direct anti-tumor effects (142), IMiDs promote immune activation including functional enhancement of T, NK, and NKT cells, increase of Th1 cytokine production, reduction of Treg activity, improvement of DC maturation and functions, and enhancement of anti-MM antibody dependent cell-mediated cytotoxicity (ADCC) (143–145). Therefore, IMiDs exert anti-angiogenic and anti-inflammatory effects and can disrupt plasma cell–BM microenvironment interactions (146). Interestingly, *in vitro* studies have demonstrated that lenalidomide treatment reduces PD-1 expression on T and NK cells and PD-L1 expression on tumor plasma cells and MDSCs (86) suggesting that IMiDs could enhance the effect of the ICP inhibitor (139). Indeed, combined therapeutic strategies with IMiDs and ICP inhibitors achieved promising results with acceptable safety and durable responses. A phase II study (NCT02289222) combining the anti-PD-1 pembrolizumab with pomalidomide and low-dose dexamethasone in 48 patients with relapsed/refractory MM resulted in an objective response of 60% including stringent complete response/complete response (8%), very good partial response (19%), and partial response (33%), with a median duration of response of 14.7 months (147). Next phase III studies of pembrolizumab in combination with pomalidomide and dexamethasone (NCT02576977) or lenalidomide and dexamethasone (NCT02579863) have been halted by the US Food and Drug Administration because of unsatisfactory results in terms of objective response and high mortality (148). A phase I trial of the anti-PD-1 nivolumab in combination with IMiDs, daratumumab, and proteasome inhibitors uncovered this combined therapy to be effective with a low toxicity profile in highly pretreated and refractory MM patients (149). Further clinical studies are ongoing to investigate the efficacy and the toxicity of nivolumab in combination with other anti-myeloma drugs in earlier stages of disease and in low-risk MM patients. In addition, clinical trials of anti-PD-L1 monoclonal antibodies (mAbs) (atezolizumab and durvalumab) alone or in combination with other anti-myeloma agents are highly expected (150).

The use of mAbs targeting CD38, daratumumab, and isatuximab is also potentially useful for treatment of relapsed/refractory MM who have received two or more prior lines of therapy (151–153). The effect of drugs alone is enhanced by the addition of IMiDs or proteasome inhibitors. Phase III trials comparing the combination of daratumumab with bortezomib and dexamethasone or lenalidomide and dexamethasone *versus* the drugs alone showed improved progression-free survival and overall response (154–157). The improvement of response and progression-free survival with acceptable safety has been recently achieved also in newly-diagnosed transplant-eligible patients by using daratumumab in combination with bortezomib, thalidomide, and dexamethasone (158).

The ICARIA-MM phase III study comparing the combination of isatuximab, pomalidomide, and dexamethasone *versus* pomalidomide and dexamethasone alone in relapsed/refractory MM patients revealed that isatuximab in the combination regimen increased the number of patients achieving a response and significantly improved the strength of response and the median progression-free survival (159).

Alternative strategies include the use of agents to disrupt BM-myeloma cell interactions. One of these agents is elotuzumab, a humanized mAb that binds to SLAMF7 (family member 7 of the signaling lymphocytic activation molecule), an immunomodulatory receptor expressed on several hematopoietic cells, including myeloma cells and NK cells (160–162). A phase I, multicenter, open-label, dose escalation study of elotuzumab showed a favorable toxicity profile but no objective responses with stable disease reported in 26% of patients (163). However, the combination of elotuzumab with pomalidomide and dexamethasone revealed a significant improvement over pomalidomide and dexamethasone alone in treatment outcomes of relapsed/refractory MM patients. Specifically, the overall response rate was higher in the elotuzumab group (53%) than in the control group (26%) with a better progression­free survival mainly observed in patients pretreated with at least four prior lines of therapy or patients who were considered as having high­risk disease on the basis of International Myeloma Working Group Criteria (164).

Immunotherapy with adoptively transferred chimeric antigen receptor (CAR) T cells targeting myeloma-associated antigens is currently being tested in clinical trials and initial results have been encouraging. A new effective therapy for MM is the use of anti-B cell maturation antigen (BCMA) CAR T cells. Treatment of relapsed/refractory MM patients provided promising results with a high overall response. However, the durability of this response was limited and even patients with initial complete response finally relapsed. Moreover, toxicities included cytokine release syndrome, and neurotoxicity has been reported (165, 166). The main mechanism of resistance to CAR T cell therapy is the evasion of fully differentiated tumor cells expressing lower levels of BCMA. Recently, the SLAM receptor CD229/LY9 has been used as potential target for chimeric antigen receptor (CAR) T cell therapy in MM due to its strong and broader expression on the surface of BM plasma cells from MM and MGUS patients and on chemotherapy-resistant myeloma precursor cells (167–169). CD229 CAR T cells displayed a strong and persistent activity against MM *in vitro* and *in vivo*. They efficiently killed not only terminally differentiated MM plasma cells, but also memory B cells and MM propagating cells (170).

Other immunotherapies including new bi-specific antibodies, which brings tumor cells into contact with immune effector cells, *e.g*., T cells and NK cells, and vaccines in combination with mAbs or checkpoint inhibitors are still in early-stage clinical trials (150). To date, bi-specific antibodies have been evaluated in relapsed/refractory MM patients with promising results (171).

## Future Directions

Our knowledge about mechanisms behind MM immunosuppression and sustenance of disease progression has advanced considerably. Crosstalk between immune cells and tumor endothelium regulates the entry and egress of immune cells within BM contributing to tumor immune surveillance and, at the same time, promoting angiogenesis, MM dissemination, and tumor growth (3, 11, 172, 173). Therefore, combination of canonical anti-angiogenesis treatments with immunomodulatory drugs may enhance the success of cancer immunotherapy. Moreover, it is clear that MM consists of several different genetic subtypes, and it is important to account for this when designing therapeutic regimens. A range of features including patient’s immune profile, patient’s baseline risk stratification, genetic mutations, disease biology, and imaging findings should be taken into account and integrated with each other to design tailored therapies targeting patients who might benefit the most from immunotherapy (174). New technologies for multidimensional measurement (for instance combination of single-cell RNA sequencing, genomic, immunophenotyping) of immune cells and proteins might help to build an “immunogram” to evaluate immune status and cancer-immune interactions in individual patients and thereby predict capacity to respond to immunotherapeutic strategies (76, 175).

Actually, along with immune-based approaches, the gene editing technology has emerged. Specifically, CRISPR-Cas9 technique can be used to detect necessary genes for MM plasma cells and genes involved in drug resistance, to explore the mechanism of drug action and to develop immunotherapy and screening for new drug targets (176).

Current research reveals that CRISPR/Cas9 is an efﬁcient gene knockout platform to improve efﬁcacy and safety of CAR T cells (177–179). Rupp et al. produced CD19-specific CAR T cells that were deficient in PD-1 using PD-1 disruption mediated by CRISPR/Cas9. The destruction of PD-1 increased CAR T cell ability to kill tumor cells *in vitro* (180). Based on these findings, CRISPR/Cas9 holds great promise for the treatment of MM.

## Author Contributions

Conceptualization: PL, AGS, and VR. Writing: PL. Data curation: PL, AGS, EM, RF, AB, FP, VDR, and AA. Funding: VR. Supervision: NS, AV, and VR. All authors contributed to the article and approved the submitted version.

## Funding

This work was supported by the Italian Association for Cancer Research (AIRC) through an Investigator Grant no. 20441 to VR. The sponsors of this study are non-profit organizations that support science in general; they had no role in gathering, analyzing, or interpreting the data.

## Conflict of Interest

The authors declare that the research was conducted in the absence of any commercial or financial relationships that could be construed as a potential conflict of interest.
